# The paradox of obesity with normal weight; a cross-sectional study

**DOI:** 10.3389/fnut.2023.1173488

**Published:** 2023-06-09

**Authors:** Yair Lahav, Aviv Kfir, Yftach Gepner

**Affiliations:** Department of Epidemiology and Preventive Medicine, School of Public Health, Sackler Faculty of Medicine, and Sylvan Adams Sports Institute, Tel-Aviv, Israel

**Keywords:** obesity, body weight, gender, body composition, body mass index

## Abstract

**Objective:**

To evaluate the prevalence of excessive adiposity among normal-weight individuals, and their cardiometabolic risk.

**Methods:**

This cross-sectional study included 3,001 participants (ages 20–95, 52% men, BMI 28.0 ± 5.5 kg/m^2^) who completed an anthropometric evaluation, dual x-ray absorptiometry (DXA) scan to measure body composition, and cardiometabolic blood markers. Excess adiposity was defined as ≥25% for men and ≥ 35% for women.

**Results:**

Of the entire study participants, 967 were in normal BMI (18.5–24.9 kg/m^2^) with a wide body fat distribution (4–49%). Of them, 26% of men and 38% of women were classified with excess adiposity. As compared to normal-weight lean participants, normal-weight obese men and women had higher triglycerides (76.5 ± 37.3 vs. 101.2 ± 50.3 mg/dL, *p* = 0.004 and 84 ± 44.2 vs. 101.4 ± 91.1 mg/dL, *p* = 0.030; respectively) and elevated low-density lipoprotein cholesterol (103.3 ± 31.7 vs. 119.6 ± 45.5 mg/dL, *p* = 0.011) and total cholesterol (171.5 ± 40.3 vs. 190.2 ± 39 mg/dL, *p* = 0.007) for men only. Among NWO, abdominal circumference was prevalent in 60% of the females with NWO (≥88 cm), but only in 4% of males (≥102 cm).

**Conclusion:**

Higher adiposity, even within normal weight, increases cardiometabolic risk, and abdominal waist circumference misclassified obesity in normal-weight individuals. This study highlights the need for a body composition evaluation to determine cardiometabolic risk for adults with normal body weight.

## Introduction

Body mass index (BMI) is a commonly used parameter for evaluating adiposity in the general population. The World Health Organization (WHO) defines overweight and obesity as abnormal or excessive fat accumulation that increases the risk of cardiometabolic disease and certain types of cancer (1). The association between BMI and all-cause mortality for BMI over 25 kg/m^2^ (overweight), and over 30 kg/m^2^ (obese), is well documented (2) and BMI has been widely used and accepted as a simple method to classify cardiometabolic risk by weight status (3). Despite being a practical tool for evaluating obesity and predicting chronic disease and mortality in a large population (4), the use of BMI to identify excess fat at the individual level has reasonable specificity but poor sensitivity, with approximately half the individuals with excessive body fat percentage (BF%), misclassified as non-obese (5, 6). A number of elements may influence the large variation of body fat mass for the same body weight, at the individual level. The main known factors are as follows: genome related issues which account for ~20% of BMI variation (7); loss of skeletal muscle mass with aging; ethnicity-related body shape and composition (8); level of exercise training, where fat-free mass is increased by resistance training and decreased by aerobic exercise (8); and somatotype, which provides a quantitative description of body shape independently of body size and can be classified as fat (endomorphy), muscular (mesomorphy), or linear (ectomorphy), (9). The phenomenon of metabolically obese normal weight individuals (NWO) was first described in the 90s (10) and these individuals represent a new category of obesity characterized by high body fat despite having a normal BMI (11). This double-edged sword combination of normal BMI and high body fat content is associated with a high risk for cardiometabolic dysregulation, metabolic syndrome, and cardiovascular risk factors (12, 13). Furthermore, a molecular review has provided evidence that white adipose tissue function is closely linked with cardiometabolic risk independent of BMI and thus contributes to a metabolically unhealthy average weight (14). High-fat content with low muscle mass may indicate normal-weight obesity syndrome and sarcopenia (15), suggesting that body composition provides more useful prognostic information about the morbidity risk of an individual than traditional proxies of adiposity such as BMI (16, 17). This large cross-sectional study was designed to determine the prevalence of NWO and to examine the relationship between NWO, NWL, and cardiometabolic risk.

## Materials and methods

### Study population

A total of 3,001 men and women who attended a nutrition clinic in the center of Israel between 2015 and 2021 were recruited for this cross-sectional study. Inclusion criteria were being above the age of 20 with any BMI (14.7 to 56 kg/m^2^). Candidates with a defibrillator device and pregnant women were excluded. The ethics committee at Tel-Aviv University approved the study protocol (0000607–3).

### Anthropometric measurements

Weight was recorded to the nearest 0.1 kg using a digital scale (SECA model 400; SECA North America) while the subjects were barefoot and dressed in shorts and a T-shirt. Height was measured to the nearest 0.5 cm by a SECA 274 Free-Standing Wireless 360 Stadiometer (SECA, Hamburg, Germany). Abdominal circumference (ABC) at the umbilicus was measured with measuring tape to the nearest 0.5 cm. Participants were instructed to exhale while standing, and an experienced research assistant made two waist measurements. A third measurement was taken if there was a disagreement (≥2 cm) between the two measurements. BMI was calculated as weight divided by the height squared (kg/m^2^).

### Blood test

Biochemical characteristics of the participants were obtained from the medical record of the healthcare services within the 6 months prior to each visit. Blood parameters included glycemic control, lipids, liver enzymes, and blood count.

### Body composition measurements

Participants were instructed to arrive at the clinic between 0700-and 1,000 after at least a 10 h fast with no prior exercise within the last 24 h (18) and were asked to wear minimal clothing (shorts and a T-shirt). All jewelry or other metal objects were removed, and participants were bladder voided before each scan. Body composition was measured from a whole-body scan conducted using a narrowed fan-beam dual X-ray Absorptiometry (DXA, Lunar Prodigy; GE Healthcare, Madison, WI), and analyzed by GE Encore 2011, ver. 13.60 software (GE Healthcare). We use a Nana protocol (16) for DXA measurement to minimize technical errors. The DXA was calibrated daily with phantoms according to the manufacturer’s guidelines. All the scans were conducted using the standard thickness mode. Subjects were centrally aligned in the scanning area, with their feet placed in custom-made foam blocks to maintain a constant distance (15 cm) between the feet for each scan. Similarly, participants’ hands were placed mid-prone with a standardized gap (3 cm) between the palms and the trunk (19).

The reliability of measurements for BF% and FFM was tested using 20 subjects who underwent DXA examination twice. The intraclass correlation coefficients (ICC) and standard error of measurement (SEM) were calculated for BF% (ICC > 0.994; SEM = 0.808%) and FFM (ICC > 0.962; SEM = 0.665 kg), indicating high reliability. These findings are consistent with previous research by Vicente-Rodríguez et al. (20) who reported that the total error of measurement (TEM) for BF% measured with DXA was below 0.5 U and *r* > 99.7.

### Statistical analysis

The normality of the distribution of continuous variables was assessed using visualization techniques: histogram and QQ plots, and by the Kolmogorov Smirnov Test. Variables with non-normal distributions were subjected to traditional transformations: square root for left-tail distributions, and log-normal transformation for right-tail distributions. Participant characteristics are presented as the mean ± SD for continuous variables, and as prevalence for categorical and dichotomic variables. Student’s t test or Pearson’s Chi-squared test were used to compare age and body anthropometrics between genders. Multivariate linear regressions were used to examine the association between cardiometabolic markers in NWL versus NWO, stratified by gender, in adjusted to age and BMI. In addition, a non-linear regression was used to analyze the relationship between BMI and BF%. Data were collected using Microsoft® Excel v.16.16.27 and analyzed using IBM® SPSS Statistics v.27.

## Results

**Figure 1:**
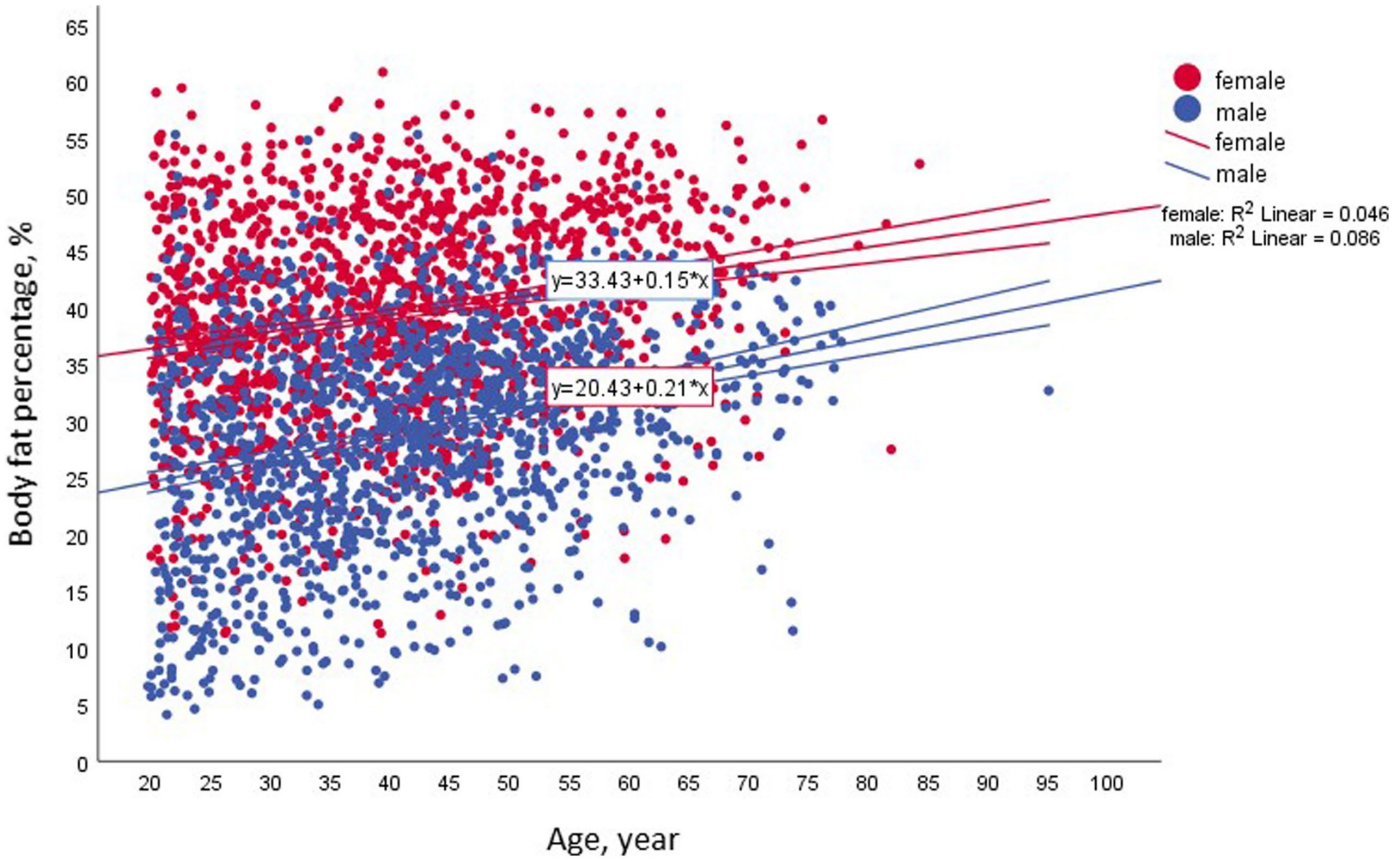
Associations between body fat and age among the entire study population across gender, *n* = 3,001.

The anthropometric characteristics of the study population across BMI categories and gender are presented in Table 1. Our study sample of 3,001 subjects comprised 1,559 (51.9%) men, and 1,442 (48.1%) women. The weight of 967 (32.2%) of the study participants was in the normal range (BMI = 18.5–24.9 kg/m^2^). These could be divided into 326 males (mean age of 36.1 ± 12 y) and 641 female (mean age of 34.7 ± 11.7 y). The association between age and BF% was modified by gender and BMI, with a low correlation of *r*^2^ = 0.024 and 0.009 for males and females, respectively (Figure 1).

**Table 1:** Study characteristics across body mass index groups and gender.

	Body mass categories											
	<18.5		18.5–24.9		25–29.9		30–34.9		35<		*p* of trend	
	F *n* = 20	M *n* = 8	F *n* = 641	M *n* = 326	F *n* = 509	M *n* = 547	F *n* = 247	M *n* = 384	F *n* = 142	M *n* = 177	F	M
BMI, kg/m^2^	17.3 ± 0.8	17.4 ± 1.3	22.5 ± 1.6	23.1 ± 1.5	27.2 ± 1.5	27.5 ± 1.4	32.2 ± 1.4	32.1 ± 1.4	39.3 ± 4.4	38.9 ± 4.2	*P* < 0.001	*P* < 0.001
Age, yr	30.8 ± 12.2	24.5 ± 3.1	34.7 ± 11.7	36.1 ± 12	41.3 ± 12.8	40.6 ± 12.2	44.3 ± 14.2	46.3 ± 12.7	45.3 ± 13.8	461 ± 12.9	*P* < 0.001	*p* < 0.001
RMR, Kcal	1,373 ± 172	1,611 ± 248	1,522 ± 176	1895 ± 254	1,613 ± 198	2031 ± 260	1732 ± 242	2,179 ± 277	1871 ± 305	2,418 ± 351	*P* < 0.001	*P* < 0.001
Height, m	1.64 ± 0.06	1.75 ± 0.11	1.65 ± 0.06	1.77 ± 0.08	1.64 ± 0.06	1.77 ± 0.07	1.63 ± 0.07	1.77 ± 0.06	1.63 ± 0.07	1.76 ± 0.07	*p* = 0.013	*p* = 0.269
Weight, kg	48.1 ± 6.3	53.6 ± 9.3	60.8 ± 6.3	72.4 ± 8.6	72.8 ± 6.4	86.3 ± 9.5	85.7 ± 8.3	101.0 ± 8.8	103.3 ± 15.1	120.4 ± 16.8	*P* < 0.001	*P* < 0.001
Body fat, %	23.6 ± 5.7	10 ± 3.8	32.3 ± 6.6	19.8 ± 7.9	41.8 ± 5.7	28.1 ± 7.0	47.9 ± 4.4	34.2 ± 5.1	50.6 ± 4.9	40.0 ± 5.3	*P* < 0.001	*P* < 0.001
Fat mass, kg	11.1 ± 5.3	5.1 ± 2.1	18.8 ± 4.8	13.7 ± 5.8	29 ± 5.0	23.2 ± 6.5	39.3 ± 4.9	33.2 ± 5.9	50.3 ± 10	46.5 ± 10.2	*P* < 0.001	*P* < 0.001
Fat free mass, kg	37 ± 4.0	48.6 ± 9.0	41.4 ± 4.9	58.7 ± 8.6	42.8 ± 5.4	62.7 ± 7.8	45.4 ± 6.1	67.3 ± 7.2	51.2 ± 7.1	72.5 ± 8.9	*P* < 0.001	*P* < 0.001
Abdominal circumference, cm	75.1 ± 7.7	74.6 ± 7.5	85.7 ± 6.3	86.9 ± 6.2	97.3 ± 6.1	98.1 ± 6.7	108.5 ± 6.6	110.1 ± 6.5	119.7 ± 11	126.0 ± 10.1	*P* < 0.001	*P* < 0.001
Neck circumference, cm	29.7 ± 1.4	34.7 ± 1.0	31.4 ± 1.6	36.4 ± 2.5	33.1 ± 1.7	39.1 ± 2.0	35.1 ± 2.6	42.1 ± 2.3	37.4 ± 3	45.0 ± 2.7	*P* < 0.001	*P* < 0.001
Glucose, mg/dl	83 ± 5.0	77 ± 6.0	87 ± 9.0	90 ± 12	90 ± 11	95 ± 16	96 ± 29	102 ± 21	99 ± 20	103 ± 18	*P* < 0.001	*P* < 0.001
Triglycerides, mg/dl	99 ± 39	70 ± 11	91 ± 68	83 ± 42	100 ± 56	119 ± 72	135 ± 88	153 ± 87	140 ± 71	170 ± 81	*P* < 0.001	*P* < 0.001
HDL-c, mg/dl	73 ± 11	51 ± 12	65 ± 15	54 ± 15	61 ± 15	49 ± 11	55 ± 12	44 ± 11	53 ± 13	42 ± 13	*P* < 0.001	*P* < 0.001
LDL-c, mg/dl	89 ± 22	92 ± 17	105 ± 33	108 ± 37	115 ± 31	117 ± 33	124 ± 34	118 ± 33	124 ± 38	113 ± 32	*P* < 0.001	*p* = 0.017
Total-c, mg/dl	182 ± 29	157 ± 25	188 ± 38	176 ± 41	194 ± 37	188 ± 39	204 ± 40	192 ± 37	205 ± 44	187 ± 34	*P* < 0.001	*p* = 0.001

Body mass categories: underweight, BMI < 18.5 (kg/m^2^); normal weight, BMI 18.5–24.9 (kg/m^2^); overweight, BMI 25–29.9 (kg/m^2^); obesity class 1, BMI 30–34.9 (kg/m^2^); obesity class 2, BMI > 35 (kg/m^2^). Values presented as mean ± SD. p of trend was tested using linear regression. F, females; M, males. Values are means ± SD.

### BMI and adiposity

**Figure 2:**
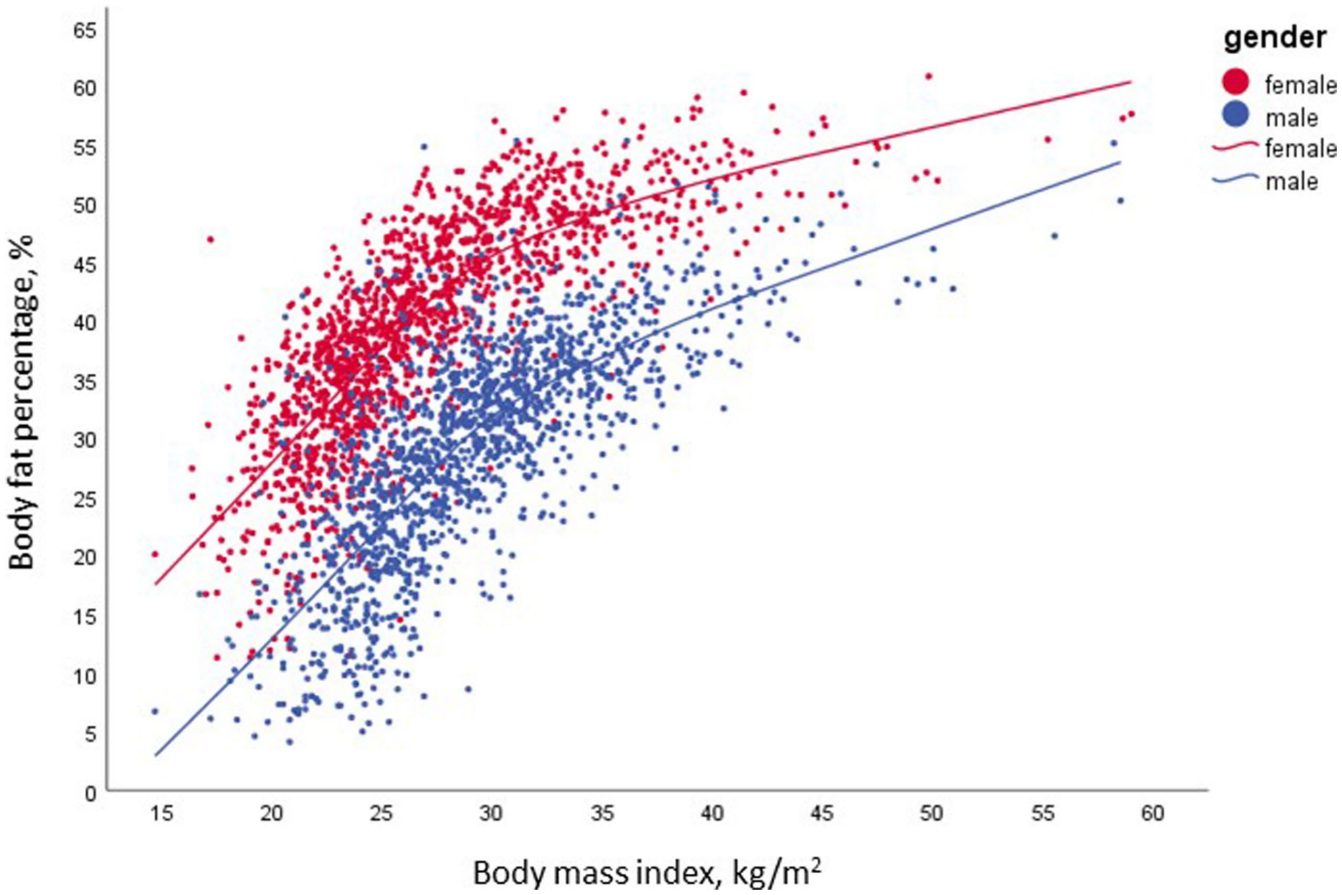
The non-linear association between BMI and fat mass; *n* = 3,001.

**Figure 3:**
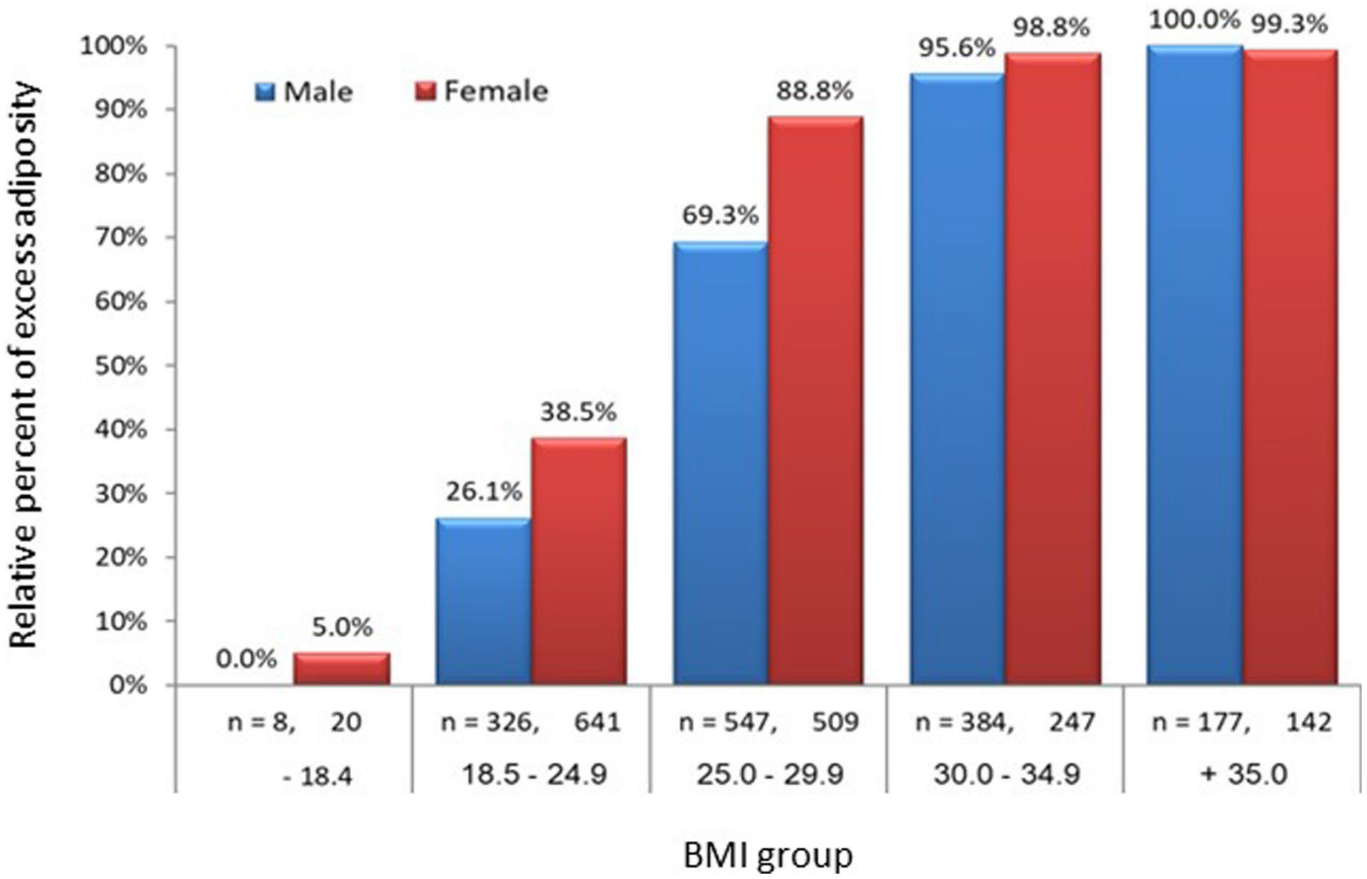
Prevalence of obesity among the study population across BMI group and gender; *n* = 3,001. For each gender, BF% was significantly increased with BMI groups. Was defined as body fat of ≥25% for men and ≥ 35% for females. Among the normal BMI group (18.5–24.9), 26% of males and 39% of females were obese.

Figure 2 shows the distribution of men and women by a combined classification of BF% across BMI categories with 25% BF (males) and 35% BF (females) used as a cutoff of excess body adiposity (21). Our results, indicate that 26% of the males and 38% of the females in the normal range of BMI (18.5–24.9 kg/m^2^), had a BF% above the cutoff. In contrast, 69.6% of males and 88.8% of females in the overweight category (BMI – 25-29.9 kg/m^2^), were above the cutoff of excess body adiposity (Figure 3).

### Abdominal circumferences vs. BF% with normal BMI

**Figure 4:**
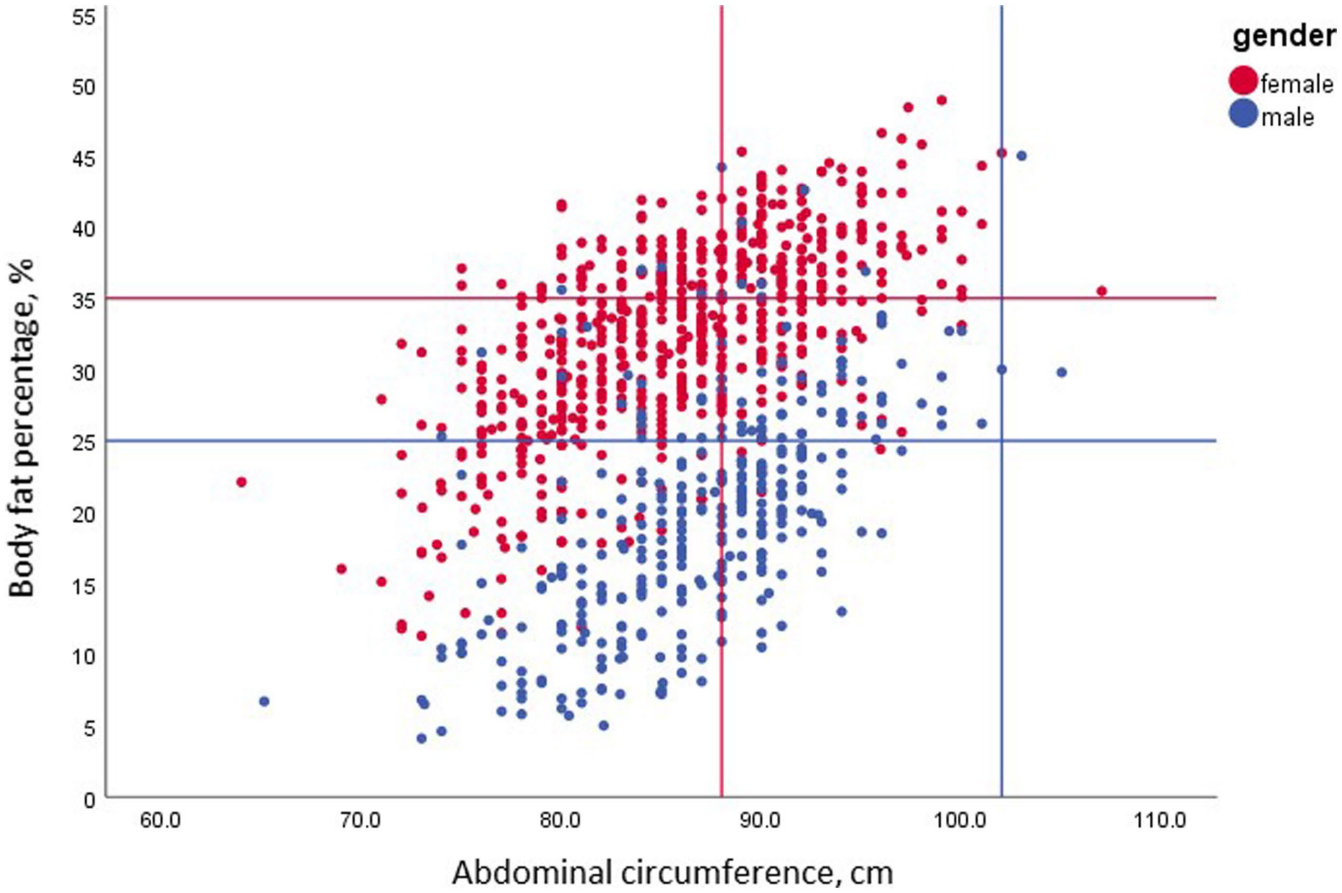
The association between body fat and elevated abdominal circumference among the normal BMI group by gender; *n* = 967. BF% in this group was strongly correlated with abdominal circumference for men (*r* = 0.61, *p* < 0.001) and women (*r* = 0.63, *p* < 0.001). However, while the clinical cutoff of 88 cm detected 60.3% of NWO females, only 3.5% of the obese males had an abdominal circumference above 102 cm.

The relationship between high BF% and large abdominal circumferences (ABC) is presented in Figure 4. Both male and female participants with normal weight (*n* = 967) exhibited a high correlation between BF% and ABC (males, *r* = 0.61, *p* < 0.001; females, *r* = 0.63, p < 0.001). Moreover, the mean ABC in NWO males was higher by 9 cm than that in the NWL counterparts. Although there was a statistically significant difference between the BMI values of the NWL and NWO groups (23.0 ± 1.59 kg/m^2^ and 23.4 ± 1.37 kg/m^2^, respectively; *p* < 0.02), this small difference cannot explain the difference in male ABC circumference, which may be attributed to the high variation in fat mass, 11.3 ± 4.1 kg in NWL versus 20.5 ± 4.7 kg in NWO individuals.

Interestingly, only 3.5% of the NWO men with BF% above 25% had an ABC above 102 cm. In contrast, the mean ABC among NWO females was higher by 6 cm than for their NWL counterparts. Again, despite the significant difference in mean BMI between NWL and NWO females (22.0 ± 1.62 kg/m^2^ and 23.1 ± 1.23 kg/m^2^, respectively; *p* < 0.02), the difference in ABC circumference can be best explained by the difference in fat mass (16.2 ± 3.8 kg in NWL and 23 ± 2.7 kg in NWO females). Notably, 39.7% of the NWO female participants had an ABC above 88 cm.

### Comparison of the clinical parameters of lean and obese normal weight participants

The characteristics of the participants belonging to two distinct body composition groups are described in Table 2. Compared to NWL males, men in the NWO group had higher triglycerides (76.5 ± 37.3 mg/dL vs. 101.2 ± 50.3 mg/dL, *p* = 0.004), LDL (103.3 ± 31.7 mg/dL vs. 107.4 ± 35.5 mg/dL, *p* = 0.011), and total cholesterol (171.5 ± 40.3 mg/dL vs. 190.2 ± 39 mg/dL; *p* = 0.007). In contrast the only statistically significant difference in females was an observation that the normal BMI group with high BF% (≥ 35%) had higher triglycerides than those with normal BMI low BF% (< 35%, 101.4 ± 91.1 vs. 84 ± 44.2 mg/dL; *p* = 0.03). We stratified the study population into different age groups (<25, 25–40, 40–60, and > 60 years) to examine the impact of age on higher adiposity in normal weight individuals and its association with cardiometabolic profile. Although the association between age groups and excess adiposity in normal weight was not statistically significant (*p* = 0.094), similar associations were found between age groups and cardiometabolic risk for each gender.

**Table 2:** The association between clinical parameters of lean vs. obese normal weight males and females.

	Males, *n* = 326		Females, *n* = 641	
	NWL	NWO	NWL	NWO
Age, y	35.5 ± 11.6	37.7 ± 13.1	37 ± 10.8	38.1 ± 12.9
Weight, kg	72.5 ± 4.4	71.8 ± 11.9	*59.6 ± 6.6	62.8 ± 5.2
BMI, kg/m^2^	*23.0 ± 1.6	23.5 ± 1.4	*22.1 ± 1.6	23.2 ± 1.2
RMR, Kcal/day	*1936 ± 240	1772 ± 268	*1539 ± 182	1,496 ± 162
Fat mass % (DXA)	*16.1 ± 5.3	29.9 ± 4.7	*28.4 ± 5.1	38.6 ± 2.7
Fat mass, kg	*11.3 ± 4.1	20.5 ± 4.7	*16.2 ± 3.8	23 ± 2.7
Fat-free mass, kg	*61.2 ± 6.8	51.3 ± 9.1	*42.9 ± 5.1	38.9 ± 3.4
Abdominal circumference, cm	*85.3 ± 5.2	91.2 ± 6.9	*83.3 ± 5.7	89.5 ± 5.3
Glucose, mg/dl	88.9 ± 11.9	91.3 ± 13.5	87.2 ± 9.5	87.8 ± 9
Triglycerides, mg/dl	*76.5 ± 37.3	101.2 ± 50.3	*84.0 ± 44.2	101.4 ± 91.1
HDL, mg/dl	54.2 ± 14.1	53.6 ± 16	65.5 ± 16.5	65.3 ± 13.9
LDL, mg/dl	*103.6 ± 31.7	119.6 ± 45.5	103.3 ± 31.7	107.4 ± 35.5
Total cholesterol, mg/dl	*171.5 ± 40.3	190.2 ± 39	186 ± 38.4	191.3 ± 37.1

Multivariate linear regressions were used to examine the association between cardiometabolic markers in NWL versus NWO, stratified by gender, and adjusted to age and BMI. Even within the normal BMI range, high body fat was associated with elevated LDL and total cholesterol among males and increased triglycerides among males and females. *p < 0.05.

## Discussion

Our study defined NWO by the coexistence of normal BMI (18.5–24.9 kg/m^2^) and excess BF% above 25% in men and 35% in females, and evaluated this phenomenon for the first time among 3,001 Israeli with a wide range of ages (20–95 y) and BMI (14.7–56 kg/m^2^). The 66.8% of the study population with BMI > 24.9 kg/m^2^ is in accordance with the WHO, estimate that 64.3% of the Israel population are overweight and obese (22). In addition, out of the 33.2% of our study population with BMI in the normal range, 26% of the men and 38% of the females could be classified as having high adiposity.

Unlike the well-defined and established BMI parameter (23), the BF% cutoff is a lesser known option for evaluating the risk of cardiometabolic diseases even in people with normal BMI. Previous studies have suggested that a BF% greater than 25% for men and 35% for women is the threshold for diagnosing obesity (24). This was adopted by the American Association of Clinical Endocrinology/American College of Endocrinology in 2004 (25). In contrast, Kim et al. (26) used a BF% threshold of ≥ 20.6% for Korean men and ≥ 33.4% for females to define NWO, while Romero-Corral et al. (12) classified 6,171 Americans as NWO by BF% > 23.1% in men and > 33.3% in women. In another study, 1,222 Brazilian aged 23–25 were defined as an NWO when the BF% exceeded > 23% in men and > 30% in women (27). Values of BF% above ≥25% for men and ≥ 35% for women were used to evaluate the combined effects of BMI and %BF% on prognosis in coronary heart disease (21). A more recent study of the threshold for BF% in the prediction of cardiovascular risk factors related to obesity concluded that, with the exception of dyslipidemia, the optimal BF% cutoff is 25.8% for men and 37.1% for women (28). Our study therefore used 25% and 35% for BF% as a cutoff for males and females to define NWO with a normal BMI, as has been used elsewhere (29–31).

It is well established that sex, age, and genetic and environmental factors all affect body fat distribution and visceral fat (32). As a result, some researchers have also suggested sex-and age-adjusted thresholds for NWO in addition to BF%: 20–39 years, > 19% and > 32%; 40–59 years, > 21% and > 33%; and 60–79 years, > 24% and > 35% for men and women, respectively (33). In our study (Figure 1), the association (R^2^) between body fat and age in the normal BMI group by gender was 0.024 for men and 0.009 for females. This finding allowed us to use the 25% and 35% thresholds for men and women, respectively, without further age division (12).

Abdominal circumference is a well-established risk factor for metabolic syndrome. An ABC above 102 cm for men and 88 cm for women has been shown to be associated with cardiovascular disease (CVD) and multiple metabolic risk factors (34). Intriguingly, in our study (Figure 4), only 3.5% of the NWO males had an elevated ABC above 102 cm. This observation questions the ability of ABC to define men with normal BMI but increased BF% as having a higher prevalence of dyslipidemia and hypertension. Similarly, Romero-Corral et al. (12) reported the presence of several metabolic abnormalities in men with normal BMI, waist circumference of 88.9 + 0.20, but elevated BF% (≥ 23.15). In contrast, the same authors (12) concluded that the mean abdominal circumference in women with a normal BMI and BF% >33.3 was 83.3 + 0.20. We emphasize that increased waist circumference was not connected to higher CV mortality, as was BF% content in subjects with NWO, and only 2% had central obesity criteria – represented by the 102 cm index. Interestingly, from our results, a cutoff of 88 cm identified 60.3% of NWO women, with the corollary that 39.7% of women had a BF% above 35%, even though their ABC was lower than 88 cm. These findings highlight the need for adiposity assessment in NWO individuals, even with normal ABC, to identify metabolic syndrome risk.

These results agree with other observations that individuals with a similar BMI may have very different metabolic and CV risk profiles (35). The complementary population to NWO individuals comprise those with “metabolically healthy obesity” (MHO), whose BMI at ≥30 kg/m^2^ would classify them as obese, but who display no other elements of metabolic syndrome. Rey-López et al. (36) reported a prevalence of MHO of between 6–74%, depending on different criteria. Longitudinal studies suggest that this phenotype may not be benign and suggest that clinicians should regard MHO as a temporary or intermediary state with the potential for individuals develop a higher risk for increased cardiovascular events and all-cause mortality over time (6). MHO is related to the absolute amount, distribution, or incorporation of fat into non-adipose tissues – referred to as ectopic fat (37). Our study found no difference in BMI between NWO and NWL (Table 2) although we did detect body composition related differences in excess fat, FFM, and ABC. We hypothesize that the metabolic consequences of cardiometabolic profiles are associated with fat mass *per se*. In support of our current observations, previous studies have also reported metabolic abnormalities in women considered NWO. For example, the prevalence of metabolic syndrome in 6171 NWO subjects(BMI = 18.5–24.9) > 20 y of age (mean age 41.3 ± 0.31) was estimated as four-fold higher than for their NWL controls (12). Similarly, analyses of BF%, BMI, and cardiovascular risk factors in a Swedish population, revealed that NWO participants had higher serum triglycerides and low-density lipoprotein cholesterol than NWL individuals (30). Korean population-based studies also detected a high prevalence of cardiometabolic abnormalities among subjects with NWO (26). In 2,078 subjects who compared NWL with NWO (cut-off, men ≥25. 4 BF% and women ≥31.4 BF%), the author showed a positive correlation between NWO and parameters such as visceral fat, fasting glucose level, and serum triglyceride level (38). Although our results revealed a significant association between NWO and a rise in TG in both genders accompanied by increases in LDL and total cholesterol in men, none of these results were high enough to be indicative of metabolic syndrome, although this could be due to age and other factors (27). In this context, a three-year cohort study (n = 190,599) reported that a 1% increase in the relative BFM increased the risk of metabolic syndrome by 24–25% (39). This result reinforces previous data presenting high blood lipids and hyperglycemia in the normal range can identify healthy persons at increased risk of developing metabolic syndrome (40).

The association between low muscle mass metabolic dysfunction and mortality is well documented (41), with the combination defined as sarcopenic obesity (42). We found a significant difference of ~10 kg in mean FFM (61.2 ± 6.8 vs. 51.3 ± 9.1) between NWL and NWO in men and ~ 4 kg (42.9 ± 5.1 vs. 38.9 ± 3.4) in women, which might emphasize the importance of preventing long term decline in physical function in this population (43). A recently published review and meta-analysis of 35 prospective cohort studies with 923,295 participants concluded that the lowest risk of all-cause mortality was observed at a BF% of 22 and 35% for men and female respectively, with a significant increase at a BF% of 27% for men and 44% for woman (44). This agrees with our findings that a BF% of 25 can be used as a reasonable cut-off for an increased risk of health problems in men.

The present study has several limitations. Primarily, as a cross-sectional investigation, it lacks the capacity to track alterations in body composition and cardiometabolic parameters over time, precluding the establishment of causality. Additionally, although the large sample size, the study was conducted solely at one research center, thereby potentially restricting the generalizability of the findings to other populations. Despite utilizing a standardized protocol for all study measurements, external validity may be limited. Furthermore, further data regarding metabolic syndrome, such as blood pressure, insulinemia, C-reactive protein, and uric acid, were not obtainable, though key cardio-metabolic markers were included in the analysis. This study possesses several strengths that warrant attention. First, the large and diverse sample of Israeli males and females, ranging from 20 to 95 years of age and presenting with a wide range of BMI values (14.7 to 56 kg/m^2^), enhances the representativeness of the findings. Moreover, unlike many prior epidemiological investigations that have employed bioelectrical impedance (BIA) for the measurement of body composition, we used DXA, a technique regarded as a gold standard in the field, to categorize individuals as having normal weight with or without excess adiposity (NWL and NWO, respectively). We have recently demonstrated that variations in the methodology employed to assess body composition can significantly impact measurement accuracy (45). Thus, our decision to use the DXA approach is noteworthy and strengthens the reliability of our results.

## Conclusion

This large cross-sectional study was designed to use the threshold values of 25% BF in men and 35% in females, measured by DXA to examine the ABC, adiposity, and CVD risks. The results indicate that higher adiposity is associated with an elevated cardiometabolic risk even for people with a normal body weight, and that ABC misclassified obesity in normal-weight people. The predictive ability of ABC as an indicator for cardiometabolic risk in individuals with normal BMI, was low, particularly in men. These results strengthen the premise that a value of BMI in the normal range, is insufficient to identify adiposity and cardiometabolic risk. The results of the study reveal that 26% of men and 38% of women normal BMI may still have the excess body fat that classifies them as being at higher risk for cardiometabolic disturbances and eventual mortality.

## Data availability statement

The original contributions presented in the study are included in the article/supplementary material, further inquiries can be directed to the corresponding author.

## Ethics statement

The Ethics Committee at Tel-Aviv University approved the study protocol (0000607–3). Written informed consent for participation was not required for this study in accordance with the national legislation and the institutional requirements.

## Author contributions

YG and YL designed research. YL and YG conducted research. YL provided essential reagents or provided essential materials. YG, AK, and YL analyzed data or performed statistical analysis. YG and YL wrote the paper. YG, AK, and YL had primary responsibility for final content. All authors contributed to the article and approved the submitted version.

## Conflict of interest

The authors declare that the research was conducted in the absence of any commercial or financial relationships that could be construed as a potential conflict of interest.

## Publisher’s note

All claims expressed in this article are solely those of the authors and do not necessarily represent those of their affiliated organizations, or those of the publisher, the editors and the reviewers. Any product that may be evaluated in this article, or claim that may be made by its manufacturer, is not guaranteed or endorsed by the publisher.
